# Development and Validation of Decision Rules to Guide Frequency of Monitoring CD4 Cell Count in HIV-1 Infection before Starting Antiretroviral Therapy

**DOI:** 10.1371/journal.pone.0018578

**Published:** 2011-04-08

**Authors:** Thierry Buclin, Amalio Telenti, Rafael Perera, Chantal Csajka, Hansjakob Furrer, Jeffrey K. Aronson, Paul P. Glasziou

**Affiliations:** 1 Division of Clinical Pharmacology and Toxicology, University Hospital Center and University of Lausanne, Lausanne, Switzerland; 2 Microbiology Institute and Swiss HIV Cohort Study board, University Hospital Center and University of Lausanne, Lausanne, Switzerland; 3 Department of Primary Health Care, Centre for Evidence-Based Medicine, University of Oxford, Oxford, United Kingdom; 4 Clinical Pharmacy Unit, Department of Pharmaceutical Sciences, University of Geneva and Lausanne, Lausanne, Switzerland; 5 University Clinic for Infectious Diseases and Swiss HIV Cohort Study Board, University Hospital and University of Bern, Bern, Switzerland; University of Cape Town, South Africa

## Abstract

**Background:**

Although CD4 cell count monitoring is used to decide when to start antiretroviral therapy in patients with HIV-1 infection, there are no evidence-based recommendations regarding its optimal frequency. It is common practice to monitor every 3 to 6 months, often coupled with viral load monitoring. We developed rules to guide frequency of CD4 cell count monitoring in HIV infection before starting antiretroviral therapy, which we validated retrospectively in patients from the Swiss HIV Cohort Study.

**Methodology/Principal Findings:**

We built up two prediction rules (“Snap-shot rule” for a single sample and “Track-shot rule” for multiple determinations) based on a systematic review of published longitudinal analyses of CD4 cell count trajectories. We applied the rules in 2608 untreated patients to classify their 18 061 CD4 counts as either justifiable or superfluous, according to their prior ≥5% or <5% chance of meeting predetermined thresholds for starting treatment. The percentage of measurements that both rules falsely deemed superfluous never exceeded 5%. Superfluous CD4 determinations represented 4%, 11%, and 39% of all actual determinations for treatment thresholds of 500, 350, and 200×10^6^/L, respectively. The Track-shot rule was only marginally superior to the Snap-shot rule. Both rules lose usefulness for CD4 counts coming near to treatment threshold.

**Conclusions/Significance:**

Frequent CD4 count monitoring of patients with CD4 counts well above the threshold for initiating therapy is unlikely to identify patients who require therapy. It appears sufficient to measure CD4 cell count 1 year after a count >650 for a threshold of 200, >900 for 350, or >1150 for 500×10^6^/L, respectively. When CD4 counts fall below these limits, increased monitoring frequency becomes advisable. These rules offer guidance for efficient CD4 monitoring, particularly in resource-limited settings.

## Introduction

The CD4 lymphocyte count, currently regarded as the best prognostic marker for the development of AIDS, is a major criterion to decide on initiation of antiretroviral therapy. In Western countries therapy for HIV-1 infection is recommended when the CD4 count falls below 350×10^6^/L, still above the cut-off of 200×10^6^/L that strongly predicts AIDS, while evidence from non-randomized studies support treatment initiation below 500×10^6^/L [1]. Other patient characteristics may encourage earlier initiation: very high counts of circulating viral particles (over 100 000 copies/mL), rapidly falling CD4 counts (more than 100×10^6^/L per year), long-lasting inflammatory symptoms or comorbidities [1]. Postponing treatment would otherwise be traditionally recommended [2]. The current trend is however to offer treatment to patients before their CD4 count reaches concerning levels, to prevent the deleterious effects of uncontrolled HIV-1 virus proliferation, which is possibly more hazardous than the albeit non-negligible adverse effects of antiretroviral drugs [3], [4], [5], [6]. In countries with limited resources, a threshold CD4 count of 200×10^6^/L is commonly used, although recent guidelines and observations also suggest earlier treatment [7], [8].

While a patient's CD4 count is above whatever threshold at which treatment will be started, repeated monitoring of the count is necessary. However, the *frequency* with which such monitoring should be undertaken is not currently clear, and monitoring strategies have not been evaluated in clinical trials. Guidelines do not give explicit recommendations about monitoring frequency during the pre-treatment phase [1], although most clinicians measure the CD4 count once every 3–6 months and some also monitor viral load. Overuse of costly determinations is undesirable. However, timely introduction of antiretroviral therapy is essential in preventing AIDS and death, and underuse of monitoring in developing countries has detrimental consequences [9], [10]. Two recent simulation studies concluded that improved HIV detection and pre-treatment CD4 monitoring in resource-poor settings could save several life-years per person taking antiretroviral treatment and could be cost-effective by preventing opportunistic infections [11], [12], [13]. Observations from the Netherlands have shown that under-screening and under-monitoring are also problematic in Western countries [14].

Our interest in evidence-based monitoring in chronic medical conditions [15] led us to analyse critically the performance of CD4 cell counts and viraemia in monitoring the pre-treatment phase of HIV infection. Our aim was to develop rational recommendations for the desirable monitoring frequency of those markers before therapy, based on published data, and to validate them in a cohort of treatment-naïve patients.

## Methods

### Literature review

We conducted a systematic review of the natural evolution and variability in CD4 cell count and viral load, to base our decision rules on the best evidence available from observational studies. Our aim was to summarize suitable descriptors of the longitudinal evolution of both these markers in HIV-infected patients followed up in prospective cohorts while receiving no antiretroviral treatments. We searched Medline and EMBASE for “CD4 OR viraemia OR viral load”, associated with “regression OR longitudinal OR slope OR monitoring”. We also examined the bibliographies of all relevant papers. The literature search was conducted in 2008 and updated in 2010. From among various statistical approaches to longitudinal analysis of HIV-1 biomarkers, we chose the most widely used and readily applicable, based on mixed-effects (multilevel) linear modelling. For CD4 counts, a majority of analyses used square-root transformation and thus only those ones were included; for viral load all used logarithmic transformation. The population parameters describing the evolution of biomarkers of HIV-1 were extracted, averaged, and rounded. Their prognostic value for the development of AIDS and prediction of death has been addressed in a large meta-analysis [16].

### Elaboration of monitoring rules

Our literature review confirmed that a mixed-effects linear model of square-root transformed CD4 counts was most often used to describe individual trajectories in untreated patients, beyond the acute changes observed during the few months after primary infection. According to this model, the square root of the CD4 count falls along a linear mean trajectory starting from a subject-specific baseline (set point a*_i_*) and is characterized by a subject-specific slope (decline rate b*_i_*); actual CD4 counts depart from this line because of random fluctuations, laboratory imprecision, and model incomplete accuracy. The subject-specific baseline (a*_i_*) and slope (b*_i_*) are considered as random variables normally distributed around average population values (α and β), while the deviations of actual CD4 counts from the subject-specific line are considered to be independently and normally distributed around zero. The subject's specific slope and intercept represent the “signal” hidden by the “noise” of within-subject fluctuations. As the fall in CD4 count depends on the individual slope and the time interval, measurements made close together capture only short-term variability and contain little information about the true slope. On the other hand, multiple determinations will refine evaluation of the subject's true current state, which may be advantageous in making therapeutic decisions, especially near the threshold for starting treatment. Monitoring decisions will therefore vary according to the distance of the patient from the threshold for antiretroviral treatment. The definition of such a threshold represents a peculiar aspect of CD4 monitoring [17].

**Figure 1:**
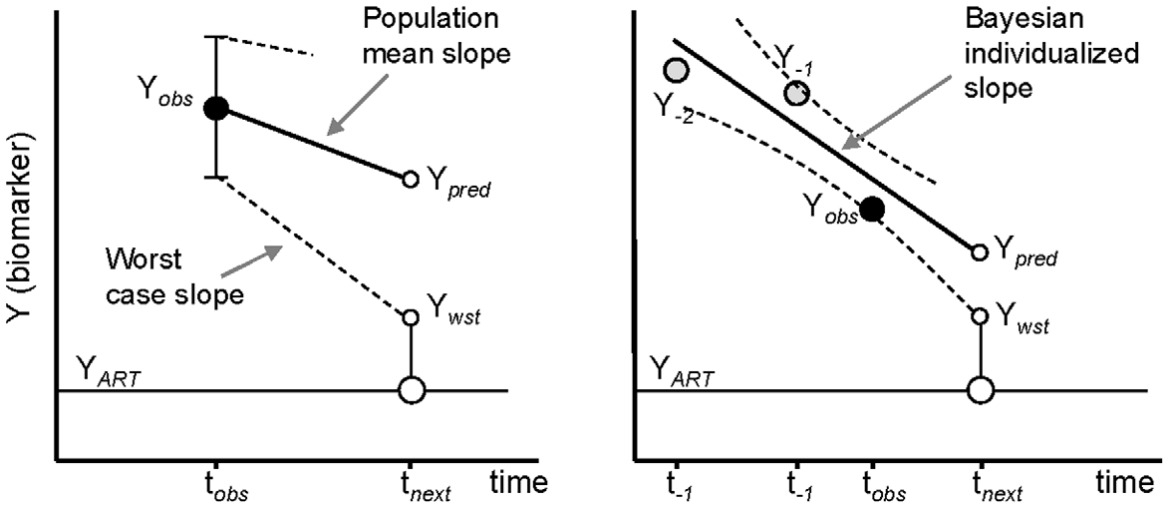
Illustration of CD4 monitoring decision rules. The *Snap-shot rule* (left) uses a single observation Y_obs_ (square root transformed) of the biomarker at time t_obs_. The *Track-shot Rule* (right) uses Y_obs_ plus one or more previous observations Y_−1_, Y_−2_ etc. available at times t_−1_, t_−2_ etc. The suitable time for next measurement, t_next_, is when the predicted value has some minimal probability *P* to reach the decision limit for antiretroviral therapy Y_ART_. Appropriate standard normal deviates z_P_ are used to weight the within-subject and between-subject dispersions, σ_e_ and σ_b_ respectively, according to the level chosen for *P*. The relevant prediction therefore depends on a worst-case scenario (Y_wst_) rather than average population prediction (Y_pred_). This illustration is schematic, as it is variances that are actually summed, not standard deviations.

We therefore designed two rules to guide decisions on CD4 monitoring frequency, detailed in Appendix S1 and illustrated in Figure 1:

***“Snap-shot rule”*** **.** The first rule applies to a *single* CD4 measurement. The aim is to determine the time to the next observation, which has a given probability of being below the decision threshold value; in other words, to determine a time at which the likelihood of finding a clinically relevant result becomes non-negligible. If the observation at this time does not reach the decision threshold, the rule can be applied recursively and will estimate shorter and shorter intervals until the rule loses its usefulness; then either the *Track-shot rule* below or frequent CD4 monitoring become necessary. The *Snap-shot rule* is thus mainly designed for relatively high CD4 cell counts.

***“Track-shot rule”*** **.** If *multiple* CD4 measurements have been performed in a subject, it may be worth using all those results to estimate the individual trajectory. A Bayesian approach is used to combine individual observations with published population estimates, directly inspired by a method developed for interpreting serum digoxin concentrations [18]. Once maximum likelihood estimates of the subject's specific slope and intercept have been determined, an approach very similar to the *Snap-shot rule* can be used to determine the suitable time for scheduling the next measurement, i.e. the nearest likely time when a new observation will reach the decision threshold with a given probability. As before, as CD4 counts come near to the decision threshold, this rule loses its usefulness and frequent CD4 monitoring becomes necessary.

Both rules can be applied either using global population values for the average baseline and slope, or taking into account individual characteristics that affect the CD4 trajectory, such as the viral load and age. The average population parameter values, their inter-individual variances, and the residual variance of square root CD4 count departures from a linear trajectory were drawn from the literature review.

Viral load is not commonly used as a biomarker for disease progression, due to prominent variability and absence of clear time trend over the asymptomatic phase of HIV-1 infection in a fair number of patients. Moreover, viral load is not usually interpreted in terms of a decision threshold for starting antiretroviral therapy. This leaves little role for specific rules to guide determination frequency, as confirmed by our attempt to apply a similar approach to this marker (Appendix S2 and Figure S1).

### Validation study

To test their performance with actual clinical data, we applied both decision rules to an unselected series of HIV-1 infected patients. The multi-centre Swiss HIV Cohort Study (SHCS), established in 1988, includes about a half of all HIV-positive individuals in Switzerland (www.SHCS.ch). Detailed clinical data are obtained from the subjects on recruitment and then at visits scheduled at least 6-monthly; all CD4 and viral load determinations are entered into the database. All patients give their written consent to epidemiological analysis of their follow up data. This validation study was approved by the SHCS scientific board.

Of the 9570 subjects recruited during the past 12 years (1 January 1996 to 31 May 2008), 5551 have undergone CD4 and viral load monitoring before starting antiretroviral therapy. The data from patients with at least *two* such recorded values were used to validate the rules outlined above, applied recursively from the first value. To prevent selection bias in favour of slow progressors, cases were included only if their first biomarker value was recorded after 1996. We did not adjust for right truncation due to treatment initiation, interruption in a subject's follow-up beyond 2008, or other causes. For all included cases, we used the rules to determine an optimal length of time between measures, based on one or more initial measures and predefined treatment decision thresholds. We started from the first CD4 count recorded, with *P* set to 0.05 (i.e. re-testing was deemed *superfluous* before having at least a 5% chance to reach the treatment initiation threshold). We also tested both rules with *P* set to 0.1. Subsequent CD4 determinations performed before the time indicated by the rule (t*_next_*) were counted, compared with the threshold, and discarded as superfluous. The *Snap-shot rule* was then reiterated on the next justifiable (i.e. non-superfluous) measurement, done after t*_next_*, and so on. The *Track-shot rule* took into account only previous CD4 values not discarded. Treatment thresholds were set at CD4 counts of 200, 350, and 500×10^6^/L.

The main objective of the study was to verify the adequacy of the classification of CD4 measures by both rules. Concretely, we aimed to check that not more than 5% of the CD4 results deemed superfluous would actually reach the threshold, using a predefined *P* level of 0.05; or not more than 10% using a *P* of 0.1. Calculations were performed using Excel (Microsoft, Redmond WA, 2003) and STATA software (v. 10, StataCorp, College-Station TX, 2007).

## Results

### Literature review

Most published descriptions of CD4 cell counts and viral load evolution in untreated HIV infection come from cohort data. We screened 149 abstracts addressing the topic of the rate of fall in CD4 counts in untreated HIV-1 infection, and we identified 40 publications that described mathematically the natural evolution of CD4 counts in untreated HIV-1 infected patients. Among them, 11 did not provide usable parameters, while 19 used longitudinal models, data transformations, or parameterizations that were not relevant to our approach (see PRISMA Flow Diagram S1). Thus, we included 8 analyses that provided suitable estimates of parameters describing the fall in CD4 count in the square-root scale according to a mixed-effects linear model [19], [20], [21], [22], [23], [24], [25], [26] (Table 1); we also included the summary estimates from two similar reviews [11], [27]. CD4 count has high variability between measures, even taken a few hours or days apart, with coefficients of variation of 13–26% [28], [29]. Square-root transformation simultaneously renders average trajectories approximately linear and residual errors approximately Gaussian, and is the method that has been used most often with CD4 count data [20], [30]. Patients are reported to differ regarding both their baseline (set point) and slope (rate of fall) of CD4 count; non-progressors remain stable over years [31] while rapid progressors lose large numbers of cells over short periods [32]. Slopes tend to be steeper in individuals who start from a high baseline, and are mainly correlated with viral load [32], [33], [34], [35] and increasing age [19], [36]; they are also affected by HIV strain [37], transmission route [38], gender [35], race [35], pregnancy [39], genetics [40], and immune reactivity [41]. However, all those factors explain only a small percentage of the overall variability in the rate of fall in CD4 count (less than 10% for viral load and age) [42]. Average CD4 decline rates have been reported to be similar between African and Western countries [43], although recent observations suggest slightly slower rates in non-white individuals [44].

**Table 1:** Published estimates of CD4 cell count decay rate and variability in untreated HIV-infected individuals.

Reference	β	σ_b_	α	σ_a_	σ_e_	ρ_ab_	N·n	duration
DeGruttola 1991 [20]	2.1	1.1	33	4	3	−0.9	495·5	3
Lange 1992 [23]	1.6	0.6	30	2	–	−0.5	327·8	3.5
Faucett 1996 [21]	2.3	2.4	25	10	2.7	−0.6	109·6	4.1
Lepri 1997 [22]	1.7	2.8°°	26	6	–	–	1021	>10
Touloumi 1998 [19]	1.4	1.2	25	5	–	–	137·9	10
Laurent 2002 [24]	1.3	–	26	–	–	–	331·3	2
CASCADE 2003 [25]	1.3 to 1.7*	1.4	23 to 29°	6	3	−0.4	5739·9	5.2
Taffé 2008 [26]	2.1	1.2	30	3	6	−0.4	4217·6	3
Phillips 2007 [27]	0 to >2†	0.8	39	2	–	–	(review)	
Hallett 2008 [11]	1.3 to 2‡	1	26	1	(50**)	–	(review)	
**Average**	**1.8**	**1.2**	**30**	**5**	**4**	**−0.5**	(rounded values)	

Parameters derived from linear mixed effect model in the square root scale. β: slope and σ_b_: inter-individual slope variability in (10^6^/L)^0.5^/year; α: intercept, σ_a_: inter-individual intercept variability and σ_e_: intra-individual variability in (10^6^/L)^0.5^; ρ_ab_: correlation between α and β; N: number of individuals, n: average number of samples per individual; average duration of observation in years.

*slope according to age (15–20: 1.30, 20–30: 1.53, 30–40: 1.73, >40: 1.67) and to symptoms at pre-infection (present: +0.26).

°intercept depending on subgroup (sex, age, intravenous drug use or haemophilia).

†slope depending on viral load (0 at <10^3^, then 0.016, 0.04, 0.12, 0.4, 0.8 and 1.6 for every 10^0.5^ step up to 2.0 at >10^6^/mL), age (+0.007/year), X4-virus shift (present: +0.25).

‡slope depending on age (<35: 1.3, >35: 2).

**standard deviation of an uniform distribution in the untransformed CD4 count scale.

°°outlier value discarded from average calculation.

There was a moderate degree of heterogeneity across the estimates shown in Table 1. To elaborate our general monitoring rules, we simply took the rounded average values. We also performed sensitivity analyses to check the robustness of the rules towards alteration in the parameters.

### CD4 monitoring recommendations

**Figure 2:**
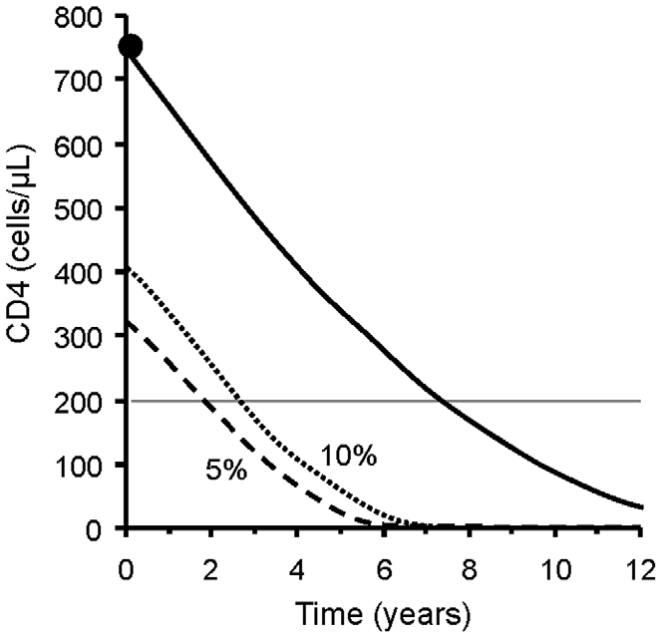
Variogram for the *Snap-shot rule*. The *Snap-shot rule* is applied to an initial CD4 cell count of 750×10^6^/L, observed at time = 0. It shows the lowest value that a subsequent measurement can be expected to reach with a probability of 5% (dashed line) or 10% (dotted line). The continuous line indicates the CD4 trajectory predicted for an average patient. The average curve will take 7.3 years to reach the 200×10^6^/L threshold (horizontal line); however, biological variability makes this outcome possible within 2 years for one patient in 20, and at 3 years for one patient in 10.

**Figure 3:**
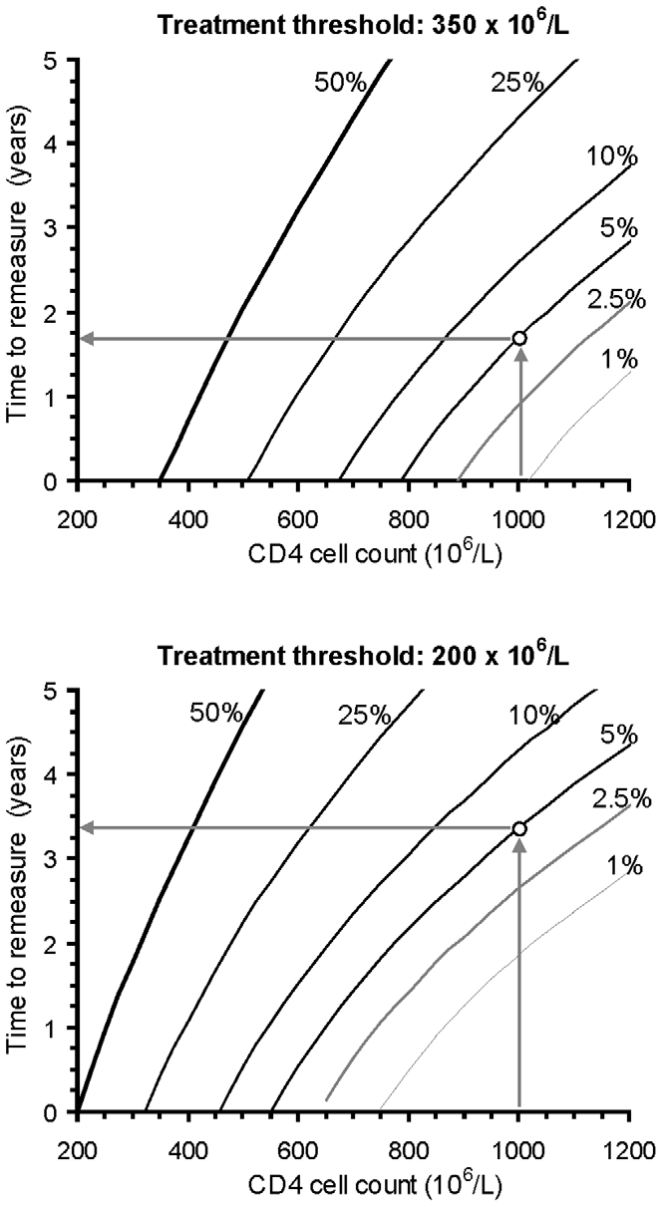
Nomograms for the *Snap-shot rule*. These nomograms show the time to wait before the next CD4 count determination as a function of the actual observation, at two decision thresholds to start antiretroviral therapy, with varying probabilities of observing a value at this threshold. The 50% lines correspond to average population predictions. The arrows illustrate the rule applied to an intial count of 1000×10^6^/L, giving about 1.7 years to reach a count of 350×10^6^/L with a 5% chance, and about 3.4 years to reach a count of 200×10^6^/L.

The operation of the *Snap-shot rule* to determine the suitable time for CD4 remeasurement is illustrated in Figure 2 using the “variogram” approach [45] based on equation 3 above. It shows the lowest CD4 count that a subsequent measurement is expected to reach with a preset probability, *P*, for a given result. The essence of this rule can be represented in a nomogram (Figure 3). The *Snap-shot rule* justifies delayed CD4 measurement for higher values observed:

- With a treatment threshold count of 200×10^6^/L, under 5% of individuals are expected to reach the threshold within 6 months after a count of 600×10^6^/L, 1 year after 650×10^6^/L, 1.5 years after 700×10^6^/L, 2 years after 770×10^6^/L, 3 years after 950×10^6^/L, or 4 years after 1100×10^6^/L.
- A threshold of 350×10^6^/L has less than a 5% chance of being reached 6 months after a value of 840×10^6^/L, 1 year after 900×10^6^/L, 1.5 years after 970×10^6^/L, 2 years after 1050×10^6^/L, or 3 years after 1250×10^6^/L.
- A threshold of 500×10^6^/L has less than a 5% chance of being reached 6 months after a value of 1060×10^6^/L, 1 year after 1140×10^6^/L, 1.5 years after 1220×10^6^/L, or 2 years after 1300×10^6^/L.

The rule loses its usefulness in the presence of low to intermediate CD4 counts (below 550, 785, and 1000×10^6^/L for the three respective decision thresholds at *P* = 0.05, and below 460, 675, and 880×10^6^/L at *P* = 0.1).

The operation of the *Track-shot rule* cannot be summarized in a chart, since it is based on serial measurements. This rule individualizes to a reasonable extent the mean expectation of the CD4 trajectory with regard to previous results. It also reduces the width of the prediction interval when many results have been recorded. But even for a trajectory defined with high precision by numerous points, this rule loses its usefulness once intra-individual variability makes it possible for the next point to reach the threshold (respectively below 430, 640, and 840 for the three thresholds at *P* = 0.05, and 370, 570, and 755 at *P* = 0.1). An Excel spreadsheet implementing both the *Snap-shot rule* and the *Track-shot rule* is available as supporting information (see Excel Tool S1).

### Validation of the CD4 monitoring rules

In total, 2608 patients from the Swiss HIV Cohort Study were entered into the validation study (Table 2). They had 18 061 CD4 measurements over a median (interquartile range, IQR) follow-up of 559 (182–1182) days, consecutive measurements being separated by 105 (85–171) days. The CD4 cell counts spread around a median of 427 (310–593)×10^6^/L, while their square-root transforms followed a fairly symmetric, bell-shaped distribution, with a mean (SD) of 20 (6.2) (cell count)^0.5^. Two thirds of the patients started antiretroviral therapy before May 2008.

**Table 2:** Description of the subpopulation of patients drawn from the Swiss HIV Cohort Study for the validation study.

Characteristic	
Number of patients	2608
Men, n (%)	1786 (68.5%)
Age, median (IQR)	35 (29–41)
Likely source of HIV infectionHeterosexual contactHomosexual contactIntravenous drug useOtherUnknown	1105 (42.4%)930 (35.7%)473 (18.1%)42 (1.6%)58 (2.2%)
Days since first HIV positive test, median (IQR)	40 (14–453)
Initial CD4 count, median×10^6^/L (IQR)	426 (286–605)
Initial viral load, median copies/mL (IQR)	24 000 (5100–93 800)
Number of CD4 determinations before antiretroviral therapy, median (IQR)	5 (3–9)
Initiation of antiretroviral therapy, n (%)	1759 (67%)
Days to initiation of antiretroviral therapy, median (IQR)	344 (87–889)
Last CD4 count before antiretroviral therapy, median×10^6^/L (IQR)	239 (166–328)

Compared with the study patients, those who could not be included because they lacked repeated CD4 testing were slightly more often women (66%, P = 0.06), older (37, IQR 32–42 years, P<0.001), and intravenous drug users (29% vs 18%, P<0.001). Above all, they had significantly lower initial CD4 counts, with a median of 250 (111–430, P<0.001), and higher initial viral loads, with a median of 51 800 (10 400–175 000, P<0.001). Among them, 2689 (91%) started antiretroviral therapy soon after their single off-treatment CD4 determination, which presumably often represented a main criterion for treatment decision.

**Figure 4:**
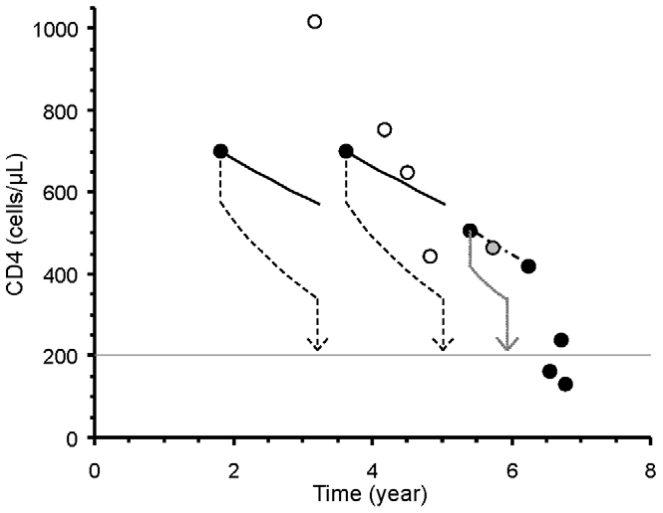
CD4 cell count data from an illustrative patient. The *Snap-shot rule* is applied to the first determination (closed circle) done 1.8 year after positive serology (discovered at time = 0), and indicates to repeat the test at 3.2 years (dashed arrow), when the CD4 count has 5% chance to reach 200 cells/µL. The second value actually measured at 3 years is thus declared superfluous and discarded (open circle). The rule is reapplied to the third value actually measured at 3.6 years, and designates the 3 next determinations as superfluous. The last 6 values fall below the threshold to question necessity. The treatment is actually initiated 7 years after HIV detection, once CD4 counts reach the threshold of 200 cells/µL. The lines indicate the average population slope. The *Track-shot rule* would give fairly similar results, except that it would indicate the 8th measurement as superfluous (dotted arrow and grey circle).

Application of the monitoring rules, illustrated in Figure 4, shows that the frequency of CD4 determinations can be kept low while high counts are observed. In most situations, the *Snap-shot rule* and the *Track-shot rule* give similar results.

We applied both rules iteratively to CD4 counts in the patients in the validation study. Table 3 summarizes the evaluation of the 15 453 non-initial CD4 values according to the rules, each run at two levels of probability (P = 0.05 and 0.1) and at three treatment decision thresholds (200, 350, and 500×10^6^/L). The *Snap-shot rule* declared fewer measurements superfluous than the *Track-shot rule* for a threshold of 200×10^6^/L, an equal amount for 350×10^6^/L, and more for 500×10^6^/L. However, the corresponding absolute counts fell dramatically on increasing the threshold, as expected. The analysis confirmed that the percentage of CD4 results that were actually below the threshold value among determinations deemed superfluous was always lower than the preset probability level *P* used to run the rules, moderately so for the 350 and 500×10^6^/L thresholds but markedly for the 200×10^6^/L threshold. There were 4905 superfluous tests in 1024 patients, taken 322 (IQR 139–605) days before the time indicated by the *Snap-shot rule* run at *P* = 0.05 for the 200×10^6^/L threshold; for the 350×10^6^/L threshold, there were 1724 superfluous tests in 421 patients, requested 287 (122–555) days in advance; and for the 500 cells/µL threshold, 625 superfluous tests in 192 patients, requested 256 (102–482) days in advance. When we restricted the analysis to the CD4 results obtained within ±3 months of the date indicated as suitable for re-measurement by the rules, the absolute numbers of values became smaller (219–2393), while the percentages of falsely superfluous CD4 results remained below the preset probability (0.68%, 2.93%, and 4.38% for the *Snap-shot rule* at *P* = 0.05 at the 200, 350, and 500×10^6^/L thresholds respectively). Very similar results emerged for the *Track-shot rule* (not detailed).

**Table 3:** Validation of the two rules on all non-initial CD4 cell counts.

Rule:			*Snap-shot rule*		*Track-shot rule*		No rule
Probability level:			*P* = 0.05	*P* = 0.1	*P* = 0.05	*P* = 0.1	
Threshold for antiretroviral therapy:	Evaluation of informativity:	Actual CD4 result:					
≤200 cells/µL	justifiable	≤200	1154	1112	1144	1069	1179
		>200	9394	7126	8268	5313	14 274
	superfluous	≤200	25	67	35	110	–
		>200	4880	7148	6006	8961	–
	*percent superfluous* *		*32%*	*47%*	*39%*	*59%*	–
	*percent ≤200 among them* °		*0.5%*	*0.9%*	*0.6%*	*1.2%*	*7.6%*
≤350 cells/µL	justifiable	≤350	5153	5108	5152	5118	5196
		>350	8576	7416	8653	7358	10 257
	superfluous	≤350	43	88	44	78	–
		>350	1681	2841	1604	2899	–
	*percent superfluous* *		*11%*	*19%*	*11%*	*19%*	–
	*percent ≤350 among them* °		*2.5%*	*3.0%*	*2.7%*	*2.6%*	*33%*
≤500 cells/µL	justifiable	≤500	9701	9661	9717	9682	9731
		>500	5127	4608	5323	4892	5722
	superfluous	≤500	30	70	14	49	–
		>500	595	1114	399	830	–
	*percent superfluous* *		*4.1%*	*7.7%*	*2.7%*	*5.7%*	–
	*percent ≤500 among them* °		*4.8%*	*5.9%*	*3.4%*	*5.6%*	*63%*

The 15 453 non-initial CD4 tests in 2608 treatment naïve patients were first classified as either justifiable or superfluous based on the rules, and then compared with the preset threshold to assess the percentage actually measured below it.

*All 95% confidence band widths <0.8% (normal approximation for binomial proportions).

°All 95% confidence band widths <1.8% (normal approximation for binomial proportions).

There was no advantage in using the rules with individualized slopes modified according to age, initial viral load, or HIV infection route (not detailed). The *Track-shot rule* evaluated average (SD) posterior slope estimates of 1.67 (0.51) (cell count)^0.5^/year, and intercept estimates of 26 (4.3) (cell count)^0.5^. Its use with a non-informative prior intercept gave very similar results; conversely, a non-informative prior slope severely compromised the validity (not detailed).

### Sensitivity analyses

Both rules were robust towards changes in parameter values, which did not markedly affect the outcomes. For the *Snap-shot rule*, a 25% reduction in the population average slope (β) translated into a few more measurements being declared superfluous (35% instead of 32% for a threshold of 250 cells/µL, 4.6% instead of 4.1% for 500 cells/µL); conversely, increasing β by 25% slightly reduced this percentage (29% instead of 32%, and 3.6% instead of 4.1%); these manipulations minimally affected the rate of falsely superfluous results (all absolute changes <0.8%). Increasing or decreasing the slope dispersion (σ_b_) by 25% had little effect on the number of either superfluous measurements (absolute changes <1.3%), or falsely superfluous results (absolute changes <0.4%). Modifying intra-patient variability (σ_e_) had more dramatic effects: a 25% reduction in σ_e_ increased both the number of superfluous measurements (47% instead of 32% for a threshold of 250 cells/µL, 8.1% instead of 4.1% for 500 cells/µL) and the rate of falsely superfluous results (0.9% instead of 0.5% for a threshold of 250 cells/µL, 5.7% instead of 4.8% for 500 cells/µL), while a 25% increase in σ_e_ had the opposite effect. Despite this, none of the actual percentages of falsely superfluous rates, calculated with a preset *P* value of 0.05, exceeded 5.7%. The *Track-shot rule* was not more sensitive to changes in the choice of parameter values (not detailed).

## Discussion

Monitoring CD4 cell counts in asymptomatic HIV-1 infected patients to decide when to start antiretroviral therapy is unanimously recommended and is cost-effective [11], [12]. However, no recommendations have yet been formulated regarding the optimal frequency of CD4 monitoring, which most UK practitioners perform every 3–4 months [46]. Based on a review of published observations describing average CD4 decline rates and variability in populations of untreated HIV-1 infected patients, we developed two decision rules aimed at guiding CD4 monitoring decisions [17]. The *Snap-shot rule* applies to a single measurement. The *Track-shot rule* takes into account a series of CD4 results in a given patient. We validated both rules using CD4 counts collected before the start of therapy in a large cohort of HIV-1 infected patients. The rules proved reliable and robust and can therefore be used to guide the management of asymptomatic patients.

We used a similar approach to evaluate the performance of repeated viral load determination based on a systematic literature review (see Appendix S2). It confirmed that this measurement has a limited role in monitoring patients who are not taking antiretroviral therapy, as others have found [12], [47].

Various approaches have been used to describe the average trend of CD4 or viral load and their inter- and intra-patient variability [48]. Since the early 1990s, mixed-effects modelling has progressively prevailed [20], [30]. Both of our rules use this approach, which minimizes contamination of the parameters that describe individual trajectories by intra-individual fluctuations and measurement errors. It appropriately shrinks the estimation of slope variability, thus clearly improving predictive performance [30].

We initially expected that the *Track-shot rule* would outperform the *Snap-shot rule*, since it adduces more information. However, except for patients starting from high CD4 levels and targeting a low treatment threshold (200×10^6^/L), the *Track-shot rule* was not superior (Table 3). Relaxing prior assumptions regarding the intercept of CD4 trajectory (α) changed nothing (though it is theoretically appropriate to do so, owing to uncertainty in the HIV-1 seroconversion date in many patients). We attribute this important finding to the fact that the fall in CD4 count with time is largely dominated by large intra-individual variability (σ_e_), which combines biological fluctuation, laboratory imprecision, and model inaccuracy; inter-individual slope variability plays the second role. This is further reflected by the results of our sensitivity analysis.

The observed rates of false superfluous CD4 tests were below the preset probability level (*P*), in particular for the low target threshold of 200×10^6^/L (Table 3). This is partly due to the more frequent repetition of CD4 testing in patients with slower rates of fall in CD4 count, while the most rapidly progressing patients were soon to receive treatment. Besides this right truncation, our validation population was also affected by selection bias. Indeed, a significant number of patients with a more active form of HIV-1 infection never underwent off-treatment CD4 monitoring, but immediately started antiretroviral therapy. This is reflected in the lower initial CD4 counts and higher viral loads in non-included patients. This is also in accordance with the fact that the post-hoc slope estimates given by the *Track-shot rule* were distributed around a lower average than we found in our literature review. Such selection bias actually represents a reassuring argument for implementing the rules in a clinical setting, where similar selection bias is likely to occur: our rules, based on average CD4 kinetic parameters, are thus expected to perform with an extra safety margin when they are used in patients whose clinical condition allows treatment to be deferred.

The rules did not perform better when age, initial viral load or HIV infection route, which are thought to modulate the average rate of fall in CD4 count, were incorporated. This is probably because, despite their statistical significance in cohort studies, age and viral load explain only a tiny part of overall CD4 variability [34], [42].

The consequences of CD4 measurement variability on therapeutic decisions were recognized in 1992 by Hoover, who suggested starting therapy after measuring two consecutive CD4 counts below the decision threshold, instead of one [28]. The phenomenon of regression to the mean, resulting from oscillation of values around their long-term trajectory, may be viewed as an argument for frequent monitoring. However, as we have shown, analysis of biological variability results in recommendations that spare measurement resources when frequent monitoring is unnecessary, in this case after a high CD4 cell count.CD4 monitoring represents only one aspect of the management of HIV-infected patients who do not require therapy, whose follow-up frequency should be arranged according to many other factors (e.g. co-morbidities, prevention of contamination, need for psychological support).

We found that roughly 11% of all CD4 cell counts performed in untreated patients were of questionable clinical usefulness, as they had less than a 5% chance of being under 350×10^6^/L. This represented 1724 measurements, or a global cost of 93 000 CHF (€58 000, £47 000, $82 000) for 421 patients. The percentage would increase to almost 40% with the *Track-shot rule* at a treatment threshold of 200×10^6^/L, which was commonly used in developing countries until very recently. Although there is no indication that our rules would perform differently in resource-poor settings, they should be validated in populations differing from Swiss HIV patients.

Finally, the main limitation of our rules is their inability to provide guidance for following CD4 cell counts when they start to approach the threshold for starting therapy. This is a consequence of the fact that the variability in CD4 count is mainly governed by the intra-individual component (σ_e_). The *Snap-shot rule* ceases to be applicable once the variogram meets the treatment threshold (Figure 2). If there are multiple CD4 determinations, the *Track-shot rule* can be used later on, but loses its usefulness once intra-individual variability makes it likely that an immediately retested CD4 count will reach the treatment threshold. Beyond this point, traditional testing at about 3 month intervals is justified. A single CD4 count below the threshold should be confirmed before starting antiretroviral therapy. In many cases will the repeat measure exceed the threshold, allowing further delay before initiation [28].

In conclusion, both theoretical arguments and observational evidence suggest that CD4 cell count monitoring, as performed in HIV-1 infected patients who do not require treatment, is currently too frequent in those whose CD4 counts are well above the treatment threshold. Infrequent measurement can safely be recommended in this subpopulation.

## Supporting Information

Figure S1 Variogram for viral load monitoring. This variogram, based on the *Snap-shot rule* for an initial determination of a viral load of 1000 copies/mL (3 log units/mL), shows the highest load that a subsequent measurement can be expected to reach, with a probability of 5% (dashed line) or 10% (dotted line). The continuous line indicates the viral load trajectory predicted in an average patient, taking about 12 years to increase by 1 log unit. After 6.7 years one patient in 20, and after 9.2 years one patient in 10, can be expected to have a 2 log unit increase (i.e. to 100 000 copies/mL, arrows).
(TIF)

Appendix S1 Construction of the monitoring rules: statistical assumptions and mathematical derivations underlying the *snap-shot rule* and the *track-shot rule*.
(PDF)

Appendix S2 Evaluation of viral load monitoring: literature review and application of the rules to viral load.
(PDF)

Excel Tool S1 An easy-to-use computer tool to assist CD4 cell count monitoring in HIV infection before starting antiretroviral therapy. It implements both the *snap-shot rule* and the *track-shot rule* to guide CD4 determination frequency.
(XLS)

PRISMA Flow Diagram S1 Outline of the literature search.
(PDF)

## References

[1] Thompson MA, Aberg JA, Cahn P, Montaner JSG, Rizzardini G (2010). Antiretroviral treatment of adult HIV infection: 2010 recommendations of the International AIDS Society-USA Panel.. JAMA.

[2] Hammer SM, Clinical practice (2005). Management of newly diagnosed HIV infection.. N Engl J Med.

[3] Phillips AN, Gazzard BG, Clumeck N, Losso MH, Lundgren JD (2007). When should antiretroviral therapy for HIV be started?. BMJ.

[4] Braithwaite RS, Roberts MS, Chang CC, Goetz MB, Gibert CL (2008). Influence of alternative thresholds for initiating HIV treatment on quality-adjusted life expectancy: a decision model.. Ann Intern Med.

[5] Kitahata MM, Gange SJ, Abraham AG, Merriman B, Saag MS (2009). Effect of early versus deferred antiretroviral therapy for HIV on survival.. N Engl J Med.

[6] Sterne JA, May M, Costagliola D, de Wolf F, Phillips AN (2009). Timing of initiation of antiretroviral therapy in AIDS-free HIV-1-infected patients: a collaborative analysis of 18 HIV cohort studies.. Lancet.

[7] World Health Organization (2006). WHO HIV/AIDS Programme: Antiretroviral therapy for HIV infection in adults and adolescents: recommendations for a public health approach.. http://www.who.int/hiv/pub/guidelines/artadultguidelines.pdf.

[8] Severe P, Jean Juste MA, Ambroise A, Eliacin L, Marchand C (2010). Early versus standard antiretroviral therapy for HIVinfected adults in Haiti.. N Engl J Med.

[9] Kent DM, McGrath D, Ioannidis JPA, Bennish ML (2003). Suitable monitoring approaches to antiretroviral therapy in resource-poor settings: setting the research agenda.. Clin Infect Dis.

[10] Kimmel AD, Losina E, Freedber KA, Goldie SJ (2006). Diagnostic tests in HIV management: a review of clinical and laboratory strategies to monitor HIV-infected individuals in developing countries.. Bull World Health Organ.

[11] Hallett TB, Gregson S, Dube S, Garnett GP (2008). The impact of monitoring HIV patients prior to treatment in resource-poor settings: insights from mathematical modelling.. PLoS Med.

[12] Bendavid E, Young SD, Katzenstein DA, Bayoumi AM, Sanders GD (2008). Cost-effectiveness of HIV monitoring strategies in resource-limited settings: a Southern African analysis.. Arch Intern Med.

[13] Egger M (2008). Antiretroviral therapy in resource-limited settings 1996 to 2006: patient characteristics, treatment regimens and monitoring in sub-Saharan Africa, Asia and Latin America.. Trop Med Int Health.

[14] Smit C, Hallett TB, Lange J, Garnett G, de Wolf F (2008). Late entry to HIV care limits the impact of anti-retroviral therapy in The Netherlands.. PLoS ONE.

[15] Glasziou PP, Irwig L, Aronson JK (2008). Evidence-based Medical Monitoring: From Principles to Practice.

[16] Egger M, May M, Chêne G, Phillips AN, Ledergerber B (2002). Prognosis of HIV-1-infected patients starting highly active antiretroviral therapy: a collaborative analysis of prospective studies.. Lancet.

[17] Stevens RJ, Oke J, Perera R (2010). Statistical models for the control phase of clinical monitoring.. Stat Methods Med Res.

[18] Sheiner LB, Beal S, Rosenberg B, Marathe VV (1979). Forecasting individual pharmacokinetics.. Clin Pharmacol Ther.

[19] Touloumi G, Hatzakis A, Rosenberg PS, O'Brien TR, Goedert JJ (1998). Effects of age at seroconversion and baseline HIV RNA level on the loss of CD4+ cells among persons with hemophilia. Multicenter Hemophilia Cohort Study.. AIDS.

[20] DeGruttola V, Lange N, Dafni U (1991). Modeling the progression of HIV infection.. J Amer Stat Assoc.

[21] Faucett CL, Thomas DC (1996). Simultaneously modelling censored survival data and repeatedly measured covariates: a Gibbs sampling approach.. Stat Med.

[22] Lepri AC, Sabin CA, Pezzotti P, England PD, Phillips AN (1997). Is there a general tendency for CD4 lymphocyte decline to speed up during human immunodeficiency virus infection? Evidence from the Italian Seroconversion Study.. J Infect Dis.

[23] Lange N, Carlin BP, Gelfand AE (1992). Hierarchical Bayes models for the progression of HIV infection using longitudinal CD4 T-cell numbers.. J Amer Stat Assoc.

[24] Laurent C, Bourgeois A, Faye MA, Mougnutou R, Seydi M (2002). No difference in clinical progression between patients infected with the predominant human immunodeficiency virus type 1 circulating recombinant form (CRF) 02_AG strain and patients not infected with CRF02_AG, in Western and West-Central Africa: a four-year prospective multicenter study.. J Infect Dis.

[25] CASCADE Collaboration (2003). Differences in CD4 cell counts at seroconversion and decline among 5739 HIV-1-infected individuals with well-estimated dates of seroconversion.. J Acquir Immune Defic Syndr.

[26] Taffé P, May M (2008). A joint back calculation model for the imputation of the date of HIV infection in a prevalent cohort.. Stat Med.

[27] Phillips AN, Sabin C, Pillay D, Lundgren JD (2007). HIV in the UK 1980–2006: reconstruction using a model of HIV infection and the effect of antiretroviral therapy.. HIV Med.

[28] Hoover DR, Graham NM, Chen B, Taylor JM, Phair J (1992). Effect of CD4+ cell count measurement variability on staging HIV-1 infection.. J Acquir Immune Defic Syndr.

[29] Raboud JM, Montaner JS, Conway B, Haley L, Sherlock C (1996). Variation in plasma RNA levels, CD4 cell counts, and p24 antigen levels in clinically stable men with human immunodeficiency virus infection.. J Infect Dis.

[30] McNeil AJ (1997). Bayes estimates for immunological progression rates in HIV disease.. Stat Med.

[31] Madec Y, Boufassa F, Porter K, Meyer L (2005). Spontaneous control of viral load and CD4 cell count progression among HIV-1 seroconverters.. AIDS.

[32] Mellors JW, Muñoz A, Giorgi JV, Margolick JB, Tassoni CJ (1997). Plasma viral load and CD4+ lymphocytes as prognostic markers of HIV-1 infection.. Ann Intern Med.

[33] Phair JP, Mellors JW, Detels R, Margolick JB, Muñoz A (2002). Virologic and immunologic values allowing safe deferral of antiretroviral therapy.. AIDS.

[34] Rodríguez B, Sethi AK, Cheruvu VK, Mackay W, Bosch RJ (2006). Predictive value of plasma HIV RNA level on rate of CD4 T-cell decline in untreated HIV infection.. JAMA.

[35] Anastos K, Gange SJ, Lau B, Weiser B, Detels R (2000). Association of race and gender with HIV-1 RNA levels and immunologic progression.. J Acquir Immune Defic Syndr.

[36] Collaborative Group on AIDS Incubation and HIV Survival including the CASCADE EU Concerted Action (2000). Time from HIV-1 seroconversion to AIDS and death before widespread use of highly-active antiretroviral therapy: a collaborative re-analysis.. Lancet.

[37] Daar ES, Kesler KL, Wrin T, Petropoulo CJ, Bates M (2005). HIV-1 pol replication capacity predicts disease progression.. AIDS.

[38] Piroth L, Binquet C, Buisson M, Kohli E, Duong M (2004). Clinical, immunological and virological evolution in patients with CD4 T-cell count above 500/mm3: is there a benefit to treat with highly active antiretroviral therapy (HAART)?. Eur J Epidemiol.

[39] Van der Paal L, Shafer LA, Mayanja BN, Whitworth JAG, Grosskurth H (2007). Effect of pregnancy on HIV disease progression and survival among women in rural Uganda.. Trop Med Int Health.

[40] Telenti A, Goldstein DB (2006). Genomics meets HIV-1.. Nat Rev Microbiol.

[41] Deeks SG, Kitchen CM, Liu L, Guo H, Gascon R (2004). Immune activation set point during early HIV infection predicts subsequent CD4+ T-cell changes independent of viral load.. Blood.

[42] Mellors JW, Margolick JB, Phair JP, Rinaldo CR, Detels R (2007). Prognostic value of HIV-1 RNA, CD4 cell count, and CD4 Cell count slope for progression to AIDS and death in untreated HIV-1 infection.. JAMA.

[43] Bakari M, Urassa W, Mhalu F, Biberfeld G, Pallangyo K (2008). Slow progression of HIV-1 infection in a cohort of antiretroviral naïve hotel workers in Dar es Salaam, Tanzania as defined by their CD4 cell slopes.. Scand J Infect Dis.

[44] May M, Wood R, Myer L, Taffe P, Rauch A (2009). CD4(+) T cell count decreases by ethnicity among untreated patients with HIV infection in South Africa and Switzerland.. J Infect Dis.

[45] Glasziou PP, Irwig L, Heritier S, Simes RJ, Tonkin A (2008). Monitoring cholesterol levels: measurement error or true change?. Ann Intern Med.

[46] Lomax N, Curtis H, Johnson M (2009). A national review of assessment and monitoring of HIV-infected patients.. HIV Med.

[47] Phillips AN, Pillay D, Miners AH, Bennett DE, Gilks CF (2008). Outcomes from monitoring of patients on antiretroviral therapy in resource-limited settings with viral load, CD4 cell count, or clinical observation alone: a computer simulation model.. Lancet.

[48] Boscardin WJ, Taylor JM, Law N (1998). Longitudinal models for AIDS marker data.. Stat Met Med Res.

