# Supplementary figures and images for: Development and Validation of Decision Rules to Guide Frequency of Monitoring CD4 Cell Count in HIV-1 Infection before Starting Antiretroviral Therapy
Source: PLoS One. 2011 Apr 8;6(4):e18578. doi: 10.1371/journal.pone.0018578 (PMC3072996; doi:10.1371/journal.pone.0018578)

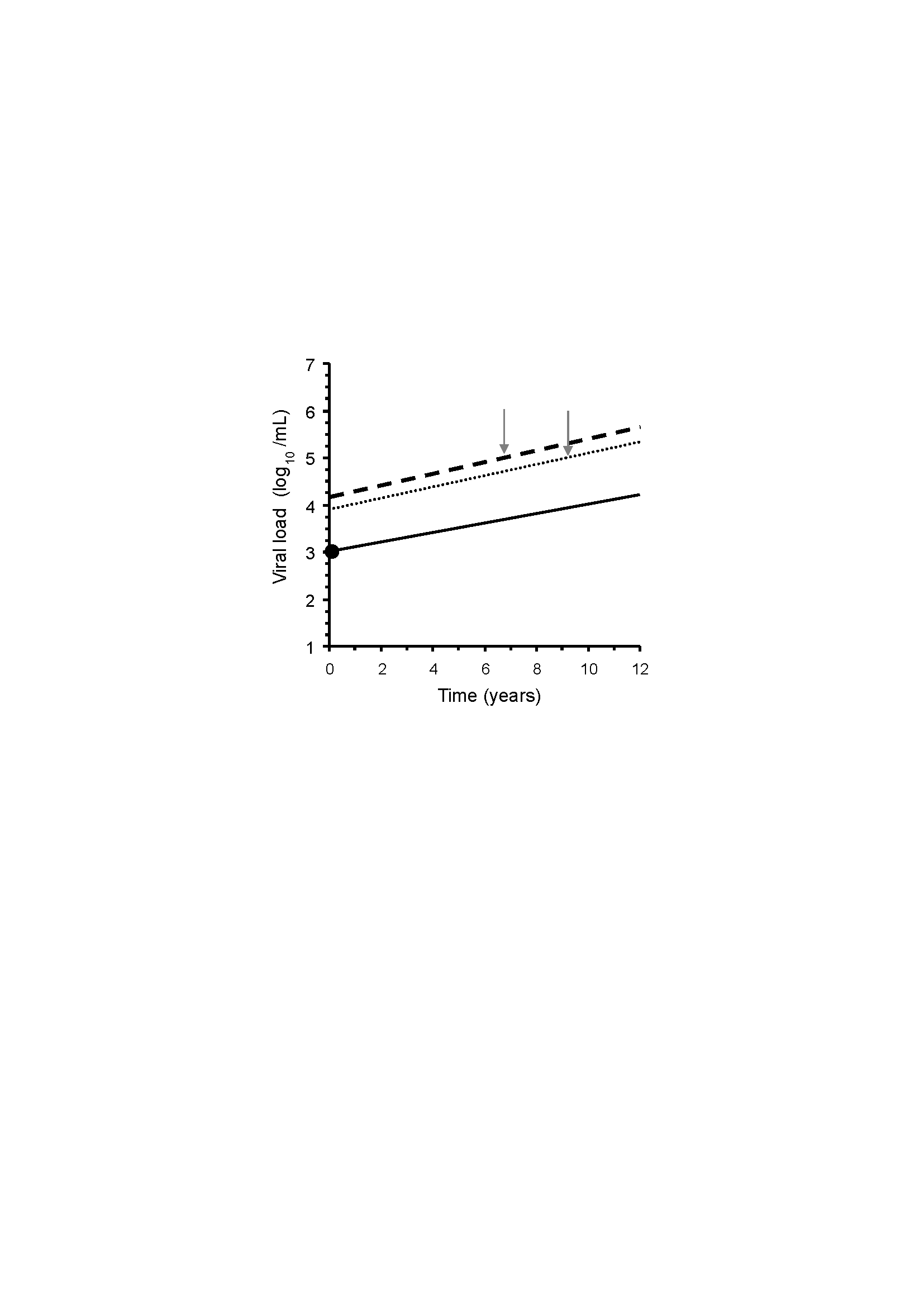

Supplement: Figure S1 — Variogram for viral load monitoring. This variogram, based on the Snap-shot rule for an initial determination of a viral load of 1000 copies/mL (3 log units/mL), shows the highest load that a subsequent measurement can be expected to reach, with a probability of 5% (dashed line) or 10% (dotted line). The continuous line indicates the viral load trajectory predicted in an average patient, taking about 12 years to increase by 1 log unit. After 6.7 years one patient in 20, and after 9.2 years one patient in 10, can be expected to have a 2 log unit increase (i.e. to 100 000 copies/mL, arrows). (TIF) [file pone.0018578.s001.tif]
