# Supplementary material for: Development and Validation of Decision Rules to Guide Frequency of Monitoring CD4 Cell Count in HIV-1 Infection before Starting Antiretroviral Therapy
Source: PLoS One. 2011 Apr 8;6(4):e18578. doi: 10.1371/journal.pone.0018578 (PMC3072996; doi:10.1371/journal.pone.0018578)
Supplement: Appendix S1 — Construction of the monitoring rules: statistical assumptions and mathematical derivations underlying the snap-shot rule and the track-shot rule. (PDF) [file pone.0018578.s002.pdf]

Supporting Document

Development and Validation of Decision Rules to Guide Frequency of Monitoring CD4 Cell Count in HIV-1 Infection before Starting Antiretroviral Therapy

Thierry Buclin, Amalio Telenti, Rafael Perera, Chantal Csajka, Hansjakob Furrer, Jeffrey K. Aronson, Paul P. Glasziou, and the Swiss HIV Cohort Study.

Appendix S1: Construction of the monitoring rules

Square-root transformation is considered appropriate for a linear description of individual trajectories of CD4 counts in untreated patients infected with HIV-1 , beyond the acute changes observed during the few months after primary infection. According to this model, the square root of the CD4 count (Y_{ij}) observed in subject *i* at time t_j can be written as:

$$Y_{ij} = a_i - b_i \cdot t_j + \varepsilon_{ij} \qquad \text{(equation 1)}$$

where a_i is the *i*th subject's specific baseline (set point) CD4 count; b_i is the slope of the fall in CD4 count; and ε_{ij} captures the within-subject variability, which is composed of short-term random fluctuation, laboratory imprecision, and model inaccuracy. The ε_{ij} values are assumed to be independent deviates sampled from a normal distribution $N(0, \sigma_e^2)$ with mean zero and standard deviation σ_e; more elaborate models allow for autocorrelation among ε_{ij} values over time. The subject-specific parameters a_i and b_i are considered to arise from population distributions $N(\alpha, \sigma_a^2)$ and $N(\beta, \sigma_b^2)$ around their respective population means α and β (with correlation ρ_{ab} unless a_i and b_i vary independently). Patient descriptors, such as age and initial viral load, can be used to refine the determination of α and β in given subgroups.

In this conceptual framework, the long-term trajectory of the individual fall in CD4 count is defined by the subject's specific slope b_i and intercept a_i, which represent the *signal* hidden by the *noise* of the within-subject fluctuations ε_{ij}. As the fall in CD4 count depends on the individual slope (b_i) and the time interval, measurements made close together capture only short-term variability and contain little information about the subject's slope. On the other hand, multiple determinations at distant intervals will refine evaluation of the subject's true trajectory and current state. Monitoring decisions will vary according to the distance of the patient from the threshold for antiretroviral treatment (ART), Y_{ART}, which represents a specific aspect of CD4 monitoring.

We therefore designed two rules to guide decisions on CD4 monitoring frequency [1], a "*snap-shot rule*" and a "*track-shot rule*":

"Snap-shot rule": The first rule applies to **a single CD4 measurement,** Y_{obs}, at time t_{obs}. Given Y_{obs}, we aim to determine the time to the next observation, Y_{next}, that has a probability P of being less than the decision threshold value Y_{ART}. This translates into determining a time, t_{next}, for which the likelihood of finding a clinically relevant result becomes non-negligible. Given Y_{obs}, the unknown true value of the subject's trajectory is contained within an interval that is determined by the within-subject variability, σ_e, the average population slope, β, and the individual slope variability, σ_b. Y_{next} can be written as:

$$Y_{next} = Y_{obs} - \beta\cdot(t_{next} - t_{obs}) + (\varepsilon_{next} - \varepsilon_{obs}) \qquad \text{(equation 2)}$$

with a mean expectation of Y_{next} given Y_{obs} :

$$E(Y_{next} \mid Y_{obs}) = Y_{obs} - \beta\cdot(t_{next} - t_{obs}) \qquad \text{(equation 3)}$$

and a variance of Y_{next} given Y_{obs} :

$$\text{var}(Y_{next} \mid Y_{obs}) = 2\cdot\sigma_e^2 + \sigma_b^2\cdot(t_{next} - t_{obs})^2 \qquad \text{(equation 4)}$$

assuming that the individual slope b and the errors ε_{next} and ε_{obs} are independent.

Based on this, a prediction interval PI_{2P} at the defined probability level $2\cdot P$ can be established around Y_{next}, using the corresponding standard normal deviate z_P:

$$PI_{2P}(Y_{next}) = \Big[\; Y_{obs} - \beta\cdot(t_{next} - t_{obs}) - z_P \cdot \sqrt{2\cdot\sigma_e^2 + \sigma_b^2\cdot(t_{next} - t_{obs})^2}\,,$$

$$Y_{obs} - \beta\cdot(t_{next} - t_{obs}) + z_P \cdot \sqrt{2\cdot\sigma_e^2 + \sigma_b^2\cdot(t_{next} - t_{obs})^2}\;\Big]$$

Thus, at a certain time t_{next}, the lower limit of this prediction interval is expected to reach the threshold value Y_{ART} with a probability P. At this time, the following relation will hold true, replacing $(t_{next} - t_{obs})$ by Δt :

$$Y_{ART} = Y_{obs} - \beta\cdot\Delta t - z_P \cdot \sqrt{2\cdot\sigma_e^2 + \sigma_b^2\cdot\ \Delta t^2} \qquad \text{(equation 5)}$$

Rearranging:

$$Y_{ART} - Y_{obs} + \beta\cdot\Delta t = - z_P \cdot \sqrt{2\cdot\sigma_e^2 + \sigma_b^2 \cdot\Delta t^2} \qquad \text{(equation 6)}$$

Squaring:

$$Y_{ART}^2 + Y_{obs}^2 + \beta^2\cdot\Delta t^2 - 2\cdot Y_{ART}\cdot Y_{obs} + 2\cdot Y_{ART}\cdot\beta\cdot\Delta t - 2\cdot Y_{obs}\cdot\beta\cdot\Delta t$$
$$= 2\cdot z_P^2\cdot\sigma_e^2 + z_P^2\cdot\sigma_b^2\cdot\Delta t^2 \qquad \text{(equation 7)}$$

Collecting the powers of Δt :

$$\Delta t^2 \cdot(\beta^2 - z_P^2\cdot\sigma_b^2) + 2\cdot\Delta t\cdot\beta\cdot(Y_{ART} - Y_{obs}) + (Y_{ART} - Y_{obs})^2 - 2\cdot z_P^2\cdot\sigma_e^2 = 0$$

(equation 8)

Solving this quadratic equation for Δt, and keeping the smaller root:

$$\Delta t = \frac{-2\cdot\beta\cdot(Y_{ART} - Y_{obs}) - \sqrt{4\cdot\beta^2\cdot(Y_{ART} - Y_{obs})^2 - 4\cdot(\beta^2 - z_P^2\cdot\sigma_b^2)\cdot(Y_{ART} - Y_{obs})^2 + 8\cdot z_P^2\cdot\sigma_e^2\cdot(\beta^2 - z_P^2\cdot\sigma_b^2)}}{2\cdot(\beta^2 - z_P^2\cdot\sigma_b^2)}$$

(equation 9)

Rearranging, simplifying, and replacing Δt with $t_{next} - t_{obs}$ finally gives the suitable time for scheduling the next measurement:

$$t_{next} = t_{obs} + \frac{-\beta\cdot(Y_{ART} - Y_{obs}) - z_P\cdot\sqrt{\sigma_b^2\cdot(Y_{ART} - Y_{obs})^2 + 2\cdot\sigma_e^2\cdot(\beta^2 - z_P^2\cdot\sigma_b^2)}}{\beta^2 - z_P^2\cdot\sigma_b^2}$$

(equation 10)

If the observation at time t_{next} does not reach the decision threshold Y_{ART}, which is expected to occur with (1 – *P*) probability, the rule can be applied recursively and will estimate shorter and shorter intervals. Once the difference ($Y_{obs} - Y_{ART}$) becomes smaller than $2\cdot z_P\cdot\sigma_e$, this rule loses its usefulness, and either the *Track-shot rule* below or frequent CD4 monitoring become necessary. The *Snap-shot rule* is thus mainly designed for relatively high CD4 cell counts.

***"Track-shot rule"*:** If **multiple CD4 measurements** have been performed in a subject, it may be worth using all those results to estimate the individual trajectory. A Bayesian approach can be used to combine individual observations with published population estimates. The overall likelihood $L_{overall}$ of the subject's specific slope b_i, intercept a_i and n individual measurements Y_j is the product of the likelihoods of each term, given by the normal probability law applied to the deviations from corresponding expectations (assuming independence between a_i, b_i, and the ε_{ij}) :

$$L_{overall} = \frac{1}{\sqrt{2\pi \cdot \sigma_b}} e^{-(b_i-\beta)^2/\sigma_b^2} \cdot \frac{1}{\sqrt{2\pi \cdot \sigma_a}} e^{-(a_i-\alpha)^2/\sigma_a^2}$$

$$\cdot \prod_{j=1}^{n} \frac{1}{\sqrt{2\pi \cdot \sigma_e}} e^{-(Y_j - a_i + b_i \cdot t_j)^2/\sigma_e^2} \qquad \text{(equation 11)}$$

After negative logarithmic transformation, the product on the right-hand side turns into a sum of squared deviations weighted by the corresponding inverse variances, corresponding to minus the log-likelihood. Thus, maximum likelihood estimates, b_i and a_i, for the subject's specific slope and intercept can be found by minimizing the following weighted sum of squares:

$$\Phi = \frac{(b_i-\beta)^2}{\sigma_b^2} + \frac{(a_i-\alpha)^2}{\sigma_a^2} + \sum_{j=1}^{n} \frac{(Y_j - a_i + b_i \cdot t_j)^2}{\sigma_e^2} \qquad \text{(equation 12)}$$

This Bayesian approach is directly inspired by the method that Sheiner and colleagues introduced for interpreting serum digoxin concentrations [2]. The first two terms express the deviation of individual parameters from average population values, inversely weighted by their known dispersion in the population; the final sum of terms expresses the deviations of actual observations from the subject's reconstructed individual curve, inversely weighted by the within-subject variability. We disregarded the correlation (ρ_{ab}) between a_i and b_i, as this term had no significant effect on the calculations. Maximum likelihood values for b_i and a_i can be obtained by setting the first partial derivatives of the formula with respect to b_i and a_i equal to zero and solving the system. An equation very similar to equation 10 can then be used to determine the suitable time for scheduling the next measurement, i.e. the nearest likely time for a new observation to reach the threshold Y_{ART} at a probability level P.

The value Y_{obs} is replaced by $Y_{pred} = a_i – b_i \cdot t_{obs}$ and the term $2 \cdot \sigma_e^2$ by $(1 + 1/n) \cdot \sigma_e^2$ to account for the improvement in the precision in Y_{pred} brought about by having *n* previous observations. If this rule is applied to a single observation it simply reduces to the *Snap-shot rule*, ignoring prior information about a_i. As before, once the difference ($Y_{obs} – Y_{ART}$) becomes smaller than $(1 + 1/n) \cdot z_P \cdot \sigma_e$, this rule loses its usefulness and frequent CD4 monitoring becomes necessary.

Both rules can be applied either using global population values for β and α or taking into account any characteristics that affect the CD4 trajectory, such as the viral load and age. The *Track-shot rule* can also be modified for a non-informative prior distribution of α (i.e. dropping the second term in eq. 6), to accommodate uncertainty about the true seroconversion date. This modification was tested as well. The parameter values (α, β, σ_a, σ_b, σ_e) were drawn from the literature review (Table 1 in the main article).

References

1. Stevens RJ, Oke J, Perera R (2010) Statistical models for the control phase of clinical monitoring. Stat Methods Med Res 19:394-414.

2. Sheiner LB, Beal S, Rosenberg B, Marathe VV (1979) Forecasting individual pharmacokinetics. Clin Pharmacol Ther 26: 294-305.
