# Supplementary material for: Development and Validation of Decision Rules to Guide Frequency of Monitoring CD4 Cell Count in HIV-1 Infection before Starting Antiretroviral Therapy
Source: PLoS One. 2011 Apr 8;6(4):e18578. doi: 10.1371/journal.pone.0018578 (PMC3072996; doi:10.1371/journal.pone.0018578)

Supporting Document

Development and Validation of Decision Rules to Guide Frequency of Monitoring CD4 Cell Count in HIV-1 Infection before Starting Antiretroviral Therapy

Thierry Buclin, Amalio Telenti, Rafael Perera, Chantal Csajka, Hansjakob Furrer, Jeffrey K. Aronson, Paul P. Glasziou, and the Swiss HIV Cohort Study.

Appendix S2: Evaluation of viral load monitoring

The use of viral load as a biomarker for HIV disease progression is less well defined than for CD4 cell count. It is not usually interpreted in terms of a decision threshold for starting antiretroviral therapy, and a fair number of patients show no detectable time trend over the whole asymptomatic phase of HIV-1 infection. We therefore limited our evaluation of viral load monitoring before treatment to general considerations based on our literature review, as a strategy based on specific rules was not thought necessary.

Literature review

For viral load progression, we were able to extract data from 6 studies [1,2,3,4,5,6] and one review [7] among 10 publications identified that described the natural evolution of CD4 counts in untreated HIV-1 infected patients (Table 1). This biomarker is highly variable, both between and within patients. The reproducibility of current assays is 0.1–0.2 $\log_{10}$ copies/mL (i.e. 25–60%) [8,9], while intra-individual variability, including biological fluctuation, is 0.26–0.31 $\log_{10}$ copies/mL [10,11,12,13]. Compared with CD4 count, viral load shows a less clear overall trend among HIV-infected individuals. Beyond the initial set point after the seroconversion peak, some authors have estimated average slopes not significantly different from zero [1,14], while others have reported a significant upward trend [2,15,16]. Viral load evolution is affected by gender [2,17,18,19], race [17], inflammatory state [20,21], and genetics [22], and correlates with the rate of fall in CD4 count and prognosis [16,23,41].

Viral load monitoring performances

A variogram after a single viral load measurement is shown in Figure 5, based on average parameters extracted from the literature. Both the mild degree of steepness expected in

the population and the importance of intra-patient variability show that early remeasurement has very little chance of capturing any significant trend beyond noise. Only after 5 years will the magnitude of the average increase equal the magnitude of intra-patient variability. This unfavourable signal/noise ratio and the absence of a clear threshold for therapeutic decision-making militate against regular viral load monitoring in patients who do not yet require antiretroviral therapy. A single measurement of viral load about 6 months after presumed seroconversion bears information about the activity of HIV infection and can influence the decision to initiate therapy in terms of CD4 threshold. Rechecking the value just before starting treatment remains important for future assessment of the virological response.

References

1. Rodríguez B, Sethi AK, Cheruvu VK, Mackay W, Bosch RJ, Kitahata M et al. (2006) Predictive value of plasma HIV RNA level on rate of CD4 T-cell decline in untreated HIV infection. JAMA. 296: 1498-1506.

2. Boscardin WJ, Taylor JM, Law N (1998) Longitudinal models for AIDS marker data. Stat Met Med Res 7: 13-27.

3. Lyles RH, Muñoz A, Yamashita TE, Bazmi H, Detels R, Rinaldo CR et al. (2000) Natural history of human immunodeficiency virus type 1 viremia after seroconversion and proximal to AIDS in a large cohort of homosexual men. Multicenter AIDS Cohort Study. J Infect Dis 181: 872-880.

4. Hubert JB, Burgard M, Dussaix E, Tamalet C, Deveau C, Le Chenadec J et al. (2000) Natural history of serum HIV-1 RNA levels in 330 patients with a known date of infection. The SEROCO Study Group. AIDS 14: 123-131.

5. Sabin CA, Devereux H, Phillips AN, Hill A, Janossy G, Lee CA, Loveday C (2000) Course of viral load throughout HIV-1 infection. J Acquir Immune Defic Syndr 23: 172-177.

6. Touloumi G, Pantazis N, Babiker AG, Walker SA, Katsarou O, Karafoulidou A et al. (2004) Differences in HIV RNA levels before the initiation of antiretroviral therapy among 1864 individuals with known HIV-1 seroconversion dates. AIDS 18: 1697-1705.

7. Phillips AN, Sabin C, Pillay D, Lundgren JD (2007) HIV in the UK 1980-2006: reconstruction using a model of HIV infection and the effect of antiretroviral therapy. HIV Med. 8: 536-546.

8. Schuurman R, Descamps D, Weverling GJ, Kaye S, Tijnagel J, Williams I et al. (1996) Multicenter comparison of three commercial methods for quantification of human immunodeficiency virus type 1 RNA in plasma. J Clin Microbiol 34: 3016-3022.

9. Chernoff DN (2002) The significance of HIV viral load assay precision: a review of the package insert specifications of two commercial kits. J Int Assoc Phys AIDS Care 1: 134-140.

10. Raboud JM, Montaner JS, Conway B, Haley L, Sherlock C, O'Shaughnessy MV, Schechter MT (1996) Variation in plasma RNA levels, CD4 cell counts, and p24 antigen levels in clinically stable men with human immunodeficiency virus infection. J Infect Dis 174:191-194.

11. Brambilla D, Reichelderfer PS, Bremer JW, Shapiro DE, Hershow RC, Katzenstein DA et al. (1999) The contribution of assay variation and biological variation to the total variability of plasma HIV-1 RNA measurements. AIDS 13: 2269-2279.

12. Paxton WB, Coombs RW, McElrath MJ, Keefer MC, Hughes J, Sinangil F et al. (1997) Longitudinal analysis of quantitative virologic measures in human immunodeficiency virus-infected subjects with > or = 400 CD4 lymphocytes: implications for applying measurements to individual patients. J Infect Dis 175: 247-254.

13. Bartlett JA, DeMasi R, Dawson D, Hill A (1998) Variability in repeated consecutive measurements of plasma human immunodeficiency virus RNA in persons receiving stable nucleoside reverse transcriptase inhibitor therapy or no treatment. J Infect Dis 178: 1803-1805.

14. Lomax N, Curtis H, Johnson M (2009) A national review of assessment and monitoring of HIV-infected patients. HIV Med 10:125-128.

15. Phillips AN, Pillay D, Miners AH, Bennett DE, Gilks CF, Lundgren JD (2008) Outcomes from monitoring of patients on antiretroviral therapy in resource-limited settings with viral load, CD4 cell count, or clinical observation alone: a computer simulation model. Lancet 371: 1443-1451.

16. O'Brien TR, Rosenberg PS, Yellin F, Goedert JJ (1998) Longitudinal HIV-1 RNA levels in a cohort of homosexual men. J Acquir Immune Defic Syndr Hum Retrovirol 18: 155-161.

17. Anastos K, Gange SJ, Lau B, Weiser B, Detels R, Giorgi JV et al. (2000) Association of race and gender with HIV-1 RNA levels and immunologic progression. J Acquir Immune Defic Syndr 24: 218-226.

18. Sterling TR, Vlahov D, Astemborski J, Hoover DR, Margolick JB, Quinn TC (2001) Initial plasma HIV-1 RNA levels and progression to AIDS in women and men. N Engl J Med 344: 720-725.

19. Napravnik S, Poole C, Thomas JC, Eron JJ (2002) Gender difference in HIV RNA levels: a meta-analysis of published studies. J Acquir Immune Defic Syndr 31: 11-19.

20. Bush CE, Donovan RM, Markowitz NP, Kvale P, Saravolatz LD (1996) A study of HIV RNA viral load in AIDS patients with bacterial pneumonia. J Acquir Immune Defic Syndr Hum Retrovirol 13: 23-26.

21. Appay V, Sauce D (2008) Immune activation and inflammation in HIV-1 infection: causes and consequences. J Pathol 214: 231-241.

22. Fellay J, Shianna KV, Ge D, Colombo S, Ledergerber B, Weale M et al. (2007) A whole-genome association study of major determinants for host control of HIV-1. Science 317: 944-947.

23. Goujard C, Bonarek M, Meyer L, Bonnet F, Chaix ML, Deveau C et al. (2006) CD4 cell count and HIV DNA level are independent predictors of disease progression after primary HIV type 1 infection in untreated patients. Clin Infect Dis 42: 709-715.

Appendix S2: Table

Published estimates of viral load increase rate and variability in untreated HIV-infected individuals

Parameters derived from linear mixed effect model in the log scale. β: slope and σ_b: inter-individual slope variability in $\log_{10}$ copies/mL/year; α: intercept, σ_a: inter-individual intercept variability and σ_e: intra-individual variability in $\log_{10}$ copies/mL; ρ_{ab}: correlation between α and β; N: number of individuals, n: average number of samples par individual; average duration of observation in years.

**Reference**	**β**	**σ_b**	**α**	**σ_a**	**σ_e**	**ρ_{ab}**	**N·n**	**duration**
Boscardin 1998 [2]	0.15	--	4	0.6	0.5	--	141·6	1.3
Hubert 1999 [4]	0.03	0.14*	4	0.8	0.5	--	330·7	4.3
Sabin 2000 [5]	0.11	--	3.3	--	--	--	105·8	>10
Lyles 2000 [3]	0.03	--	4.5	0.5*	--	--	4	3
Touloumi 2004 [6]	0.1 °	0.13	3.3 to 4.4†	1	--	-0.14†	1864·5	4.3
Rodríguez 2006 [1]	0.06	0.08*	--	--	--	--	1289·8	3
Phillips 2007 [7]	0.11 to 0.21‡	0.3	4	0.5	--	--‡	(review)	
**Average**	**0.1**	**0.15**	**4**	**0.7**	**0.5**	**0**	(rounded value)	

Parameters derived from linear mixed effect model in the log scale. β: slope and σ_b: inter-individual slope variability in $\log_{10}$ copies/mL/year; α: intercept, σ_a: inter-individual intercept variability and σ_e: intra-individual variability in $\log_{10}$ copies/mL; ρ_{ab}: correlation between α and β; N: number of individuals, n: average number of samples par individual; average duration of observation in years.

* half interquartile range
° higher in case of symptoms on seronconversion (present: 0.15)
† intercept depending on subgroup (sex, age, intravenous drug use or haemophilia)
‡ slope depending on initial viral load (0.11 at <10^3, then 0.13, 0.14, 0.15, 0.17, 0.18 and 0.2 for every $10^{0.5}$ step up to 0.21 at >10^6/mL)

Appendix S2: Figure

Variogram for viral load monitoring

This variogram, based on the *Snap-shot rule* for an initial determination of a viral load of 1000 copies/mL (3 log units/mL), shows the highest load that a subsequent measurement can be expected to reach, with a probability of 5% (dashed line) or 10% (dotted line). The continuous line indicates the viral load trajectory predicted in an average patient, taking about 12 years to increase by 1 log unit. After 6.7 years one patient in 20, and after 9.2 years one patient in 10, can be expected to have a 2 log unit increase (i.e. to 100 000 copies/mL, arrows).

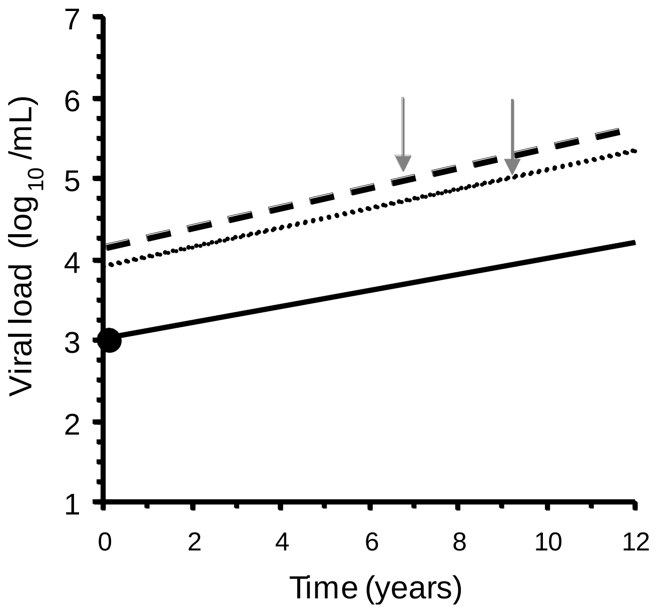

Supplement: Appendix S2 — Evaluation of viral load monitoring: literature review and application of the rules to viral load. (PDF) [file pone.0018578.s003.pdf]
