# Supplementary material for: Development and Validation of Decision Rules to Guide Frequency of Monitoring CD4 Cell Count in HIV-1 Infection before Starting Antiretroviral Therapy
Source: PLoS One. 2011 Apr 8;6(4):e18578. doi: 10.1371/journal.pone.0018578 (PMC3072996; doi:10.1371/journal.pone.0018578)
Supplement: PRISMA Flow Diagram S1 — Outline of the literature search. (PDF) [file pone.0018578.s005.pdf]

PRISMA 2009 Flow Diagram

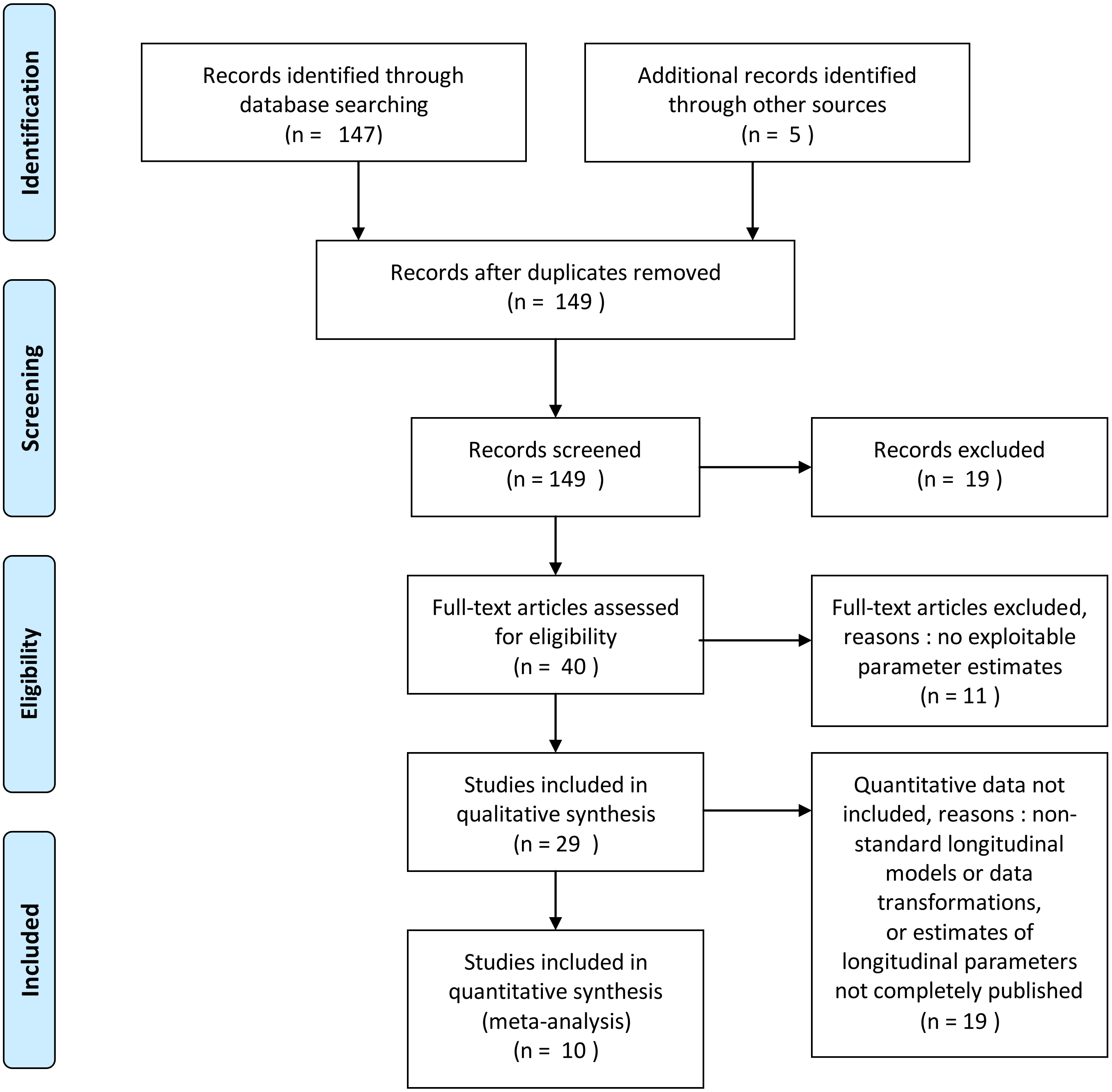

From: Moher D, Liberati A, Tetzlaff J, Altman DG, The PRISMA Group (2009). *P*referred *R*eporting *I*tems for *S*ystematic Reviews and *M*eta-*A*nalyses: The PRISMA Statement. PLoS Med 6(6): e1000097. doi:10.1371/journal.pmed1000097

For more information, visit www.prisma-statement.org.
